# Retrospective judgments of confidence in a complex span task

**DOI:** 10.1038/s41598-023-45552-y

**Published:** 2023-10-28

**Authors:** Giovanny Lau, Chris J. A. Moulin, Sophie Portrat

**Affiliations:** Univ. Grenoble Alpes, Univ. Savoie Mont Blanc, CNRS, LPNC, 38000 Grenoble, France

**Keywords:** Psychology, Human behaviour

## Abstract

Although the study of metamemory monitoring originated in predictions for simple span tasks, the study of metacognition for working memory (WM) has been somewhat neglected in comparison with long-term memory. We aimed to fill this gap by exploring the ability to self-assess WM operations. Thirty-four participants performed 16 series of complex span tasks and rated their confidence in a verbal recall paradigm. We manipulated the cognitive load based on the TBRS model in order to analyze the role of attentional resources on both WM and metacognitive evaluations. As expected, we found that recall is affected by cognitive load and we found standard serial position effects. Interestingly, metacognitive evaluations followed the same pattern, and measures of metacognitive sensitivity suggest that participants are able to make item-by-item retrospective judgments reflective of their performance. We discuss how these results contribute to our understanding of metacognitive access to newly-formed WM contents.

## Introduction

Working memory (WM) operates to temporarily maintain and manipulate information in the service of action and cognition. It is considered a central component for cognitive functioning. The extent to which this cognitive component can be metacognitively accessed is not well established. Although metacognition has been extensively studied in emotions^1^, attention^2^, reasoning^3^, language^4^, motor tasks^5^, perception^6^, decision making^7^, and long-term memory (LTM)^8^; metacognition for WM has been somewhat neglected. This is most evident in comparison with the extensive literature on metacognition for LTM (see^9^ for a review).

Establishing the extent to which WM is under metacognitive control is of critical interest for understanding the function of WM including in applied contexts but also for our understanding of the cognitive architecture of metacognition (see^10^ for an examination of how metacognition might be organized across different cognitive domains).

There is a diverse set of theories in WM (for review see^11^), yet a common idea is that the information held in WM can be manipulated and temporarily stored to support action and cognition^12–14^. Such simultaneous manipulation and storage imply strategies implementation and resource allocation which presumably depends on evaluating function in line with current goals. Indeed, a metacognitive approach to memory (e.g.,^15^) typically sees it as a system whereby monitoring of cognitive processes enables efficient regulation through appropriate control, with monitoring and control operating as a servo system, but such a framework is mostly discussed with reference to LTM.

Previous research on the relationships between WM and metacognition can be divided into two types: research into WM mechanisms in metacognitive processes, and research featuring direct self-evaluations of WM function. Firstly, researchers have considered that WM mechanisms are crucial for performing metacognitive evaluations. Nelson and Narens^15^ proposed that LTM monitoring per se occurs in WM “by copying LTM information into WM” (p.134,^15^). Thus, if WM is compromised, metacognitive performance might be inappropriate when the information to be monitored is not consolidated in LTM.

For instance, delayed predictions in LTM tasks are more accurate than immediate predictions^16–18^ and vividness ratings of WM content are more affected by dual tasks when stimuli are nonsensical (i.e., when the information is not consolidated in LTM)^19^. Moreover, active manipulation of WM contents can impair metacognition for perceptual tasks^20^, and clarity of content in visual WM can impact metacognitive parameters^21^. In fact, it has been suggested that there is an "entanglement between metacognition and WM” (p.568,^22^).

Overall, this first group of studies indicates a relationship between WM and metacognition, highlighting the crucial role of WM in metacognitive monitoring. Specifically, metacognitive monitoring is proposed to rely inherently on access to WM content, particularly in the context of LTM tasks, since evaluations are presumably performed on the material held in WM. However, it is essential to elucidate the functional disparities between WM and LTM, as these disparities can manifest in participants’ performance and maintenance strategies^23–27^. Indeed, monitoring in the context of a metacognitive task on LTM information and monitoring newly-formed information held in WM must differ from each other.

Whereas WM can be thought of as an essential component in holding in mind information for which we want to make a metacognitive judgment, as implied in theoretical accounts of metacognition, here our emphasis is more directly on the appraisal of the information held in WM in the context of a relatively process-pure WM. Importantly, recent research has directly addressed this question, yielding valuable outcomes. This second group of studies considers the measurement of subjects’ evaluations of their WM function. This approach allows for the examination of metacognitive monitoring by determining whether individuals' metacognitive evaluations align appropriately with their objective performance in typical WM tasks.

Metacognitive monitoring is shown where people’s metacognitive evaluations are appropriate given their actual performance. For this purpose, in simple span tasks, metacognitive bias and sensitivity have been measured to determine metacognitive function. Bias refers to the difference in magnitude between confidence and performance, in the sense of being under or overconfident. Sensitivity indicates the extent to which metacognitive evaluations reflect the participant’s ability to distinguish their correct from incorrect responses in their metacognitive evaluations^28–30^.

In one of the earliest studies that explored access to WM, Murphy et al.^31^ explored whether participants could predict their performance in a serial recall task inspired by Flavell’s pioneering work in metamemory (e.g.,^32^). In their study, participants estimated their span (i.e., the number of items they could successfully recall) for a set of line drawings. They were presented with two items and asked *“could you remember this many?”*. A longer list was presented each time the participant reported that they could remember the list, with the estimated span being the longest list that the participant predicted to be able to remember. After this prediction phase, participants carried out a span task. The primary aim of this task was to compare younger and older adults, in order to see if metamemory function could explain memory dysfunction in the older group, but no differences were found between groups in the accuracy of their predictions.

Metacognitive judgments map onto robust effects observed in simple-span tasks, such as the serial position effect (see^33–36^ for reviews of the serial position effect). It has also been observed that predictions of capacity in simple-span tasks with words vary before and after the encoding phase and depending on the nature of the word to be recalled (i.e., longer, shorter, similar, or dissimilar)^37^. In other words, metacognitive judgments are affected by the word-length and phonological similarity effects showing an analogous pattern to recall (see^38^). Likewise, predictions of capacity in simple-span tasks with images vary before and after the encoding phase. For instance, Bertrand et al.^39^ observed that predictions after the encoding phase showed a lower metacognitive bias than predictions made before the encoding phase. Neuropsychological data show that patients suffering from Alzheimer’s Disease seem capable of adjusting their metacognitive evaluations for image span tasks and are as accurate as healthy older adults^40^. These outcomes suggest an updating of metacognitive evaluations following the encoding experience. However, the problem with the use of images (of objects) and (real) words in these studies is that they encourage strategies that broadly involve LTM knowledge.

It is worth noting that these previous studies used either only global judgments or a variation of them. Global judgments are used to study the ability to evaluate the general performance for a task, unlike local judgments which are made on each item and allow a more fine-grained analysis of monitoring^41,42^. The problem is that global predictions are limited and allow the measurement of only metacognitive bias while neglecting sensitivity^43,44^. Furthermore, since global judgments evaluate the whole performance, they tend to target WM capacity rather than an awareness of the current contents of WM. The difference between capacity and content is crucial. WM capacity can be referred as the capacity to maintain a certain amount of information^23,25,45^. In contrast, WM content refers to the representations in WM that are supposed to “be directly accessible for conscious inspection” (p.510,^46^). Thus, global judgments of simple span tasks might be inadequate to investigate metacognitive access to WM representations themselves.

From a different approach, Reyes and Sackur^47^ provide relevant findings highlighting the ability to introspect on contents in WM. In their study, participants performed a probe recognition-based task within a simple span paradigm and estimated the number of items they needed to scan after identifying the probe. The researchers manipulated retrieval instructions, prompting participants to identify recent items relative to a distractor or determine item presence. Notably, both performance and estimations varied similarly depending on the retrieval instructions, with the identification of recent items being strongly influenced by serial position. Crucially, the subjective evaluation of how far back the participant needed to scan WM to identify a target was related to the actual serial position, suggesting access to information in WM for such introspective evaluations.

To investigate metacognitive access to WM representations, local judgments appear to be the most appropriate method. Retrospective judgments of confidence are typically used as item-by-item judgments (i.e., local judgments.,^48^). Access to metacognitive awareness of the ongoing task is described as second-order performance, and the task itself as first-order performance^49^. Sahar et al.^50^ used such a procedure; asking participants to memorize images presented one by one at different locations. Participants performed a spatial recognition of a probed item and then rated their confidence in the item’s location choice. In a second experiment, participants had to respond first to whether the probed item was displayed, then rate their confidence, and finally perform the location judgment. They also contrasted real objects with distorted objects. Overall, the results showed moderate metacognitive sensitivity to visual WM performance and a solid serial position effect on WM and confidence.

Inspired by this approach, we were motivated to explore the same issues for verbal WM within a serial recall paradigm. Specifically, we aimed to determine whether people have metacognitive access to their WM content within a complex span task. To better investigate metacognitive awareness of ongoing processes, we also manipulated cognitive load (CL). According to the time-based resources sharing (TBRS) model^13^, WM relies on a sole and limited attentional resource, so when attention is occupied by information processing, it is no longer available to maintain information, and the activation of the items to be memorized decreases, which translates into a gradual erasure of the trace^51–53^. CL is defined by the proportion of time during which attention is captured by the processing task. Thus, higher CL negatively affects the WM performance calculated as the number of items recalled^54,55^.

In our experiment, for each trial, participants memorized a series of six letters while performing a processing task after each single letter. The processing task was performed on digits for which the participant had to judge either their location on the screen or whether they were odd or even. These two tasks resulted in a low and a high CL condition respectively^56^. We calculated two WM scores (a) item recall and (b) strict recall. Item recall scored as correct the items recalled regardless of their original position, whereas strict recall counted as correct only the items recalled in their original position. Immediately after recall, each participant performed two item-by-item judgments of confidence regarding (a) only the retrieved item and (b) the retrieved item and its position. This allowed us to evaluate metacognitive sensitivity for the two different WM measures.

We expected to reproduce the effects of CL and serial position on WM scores. Most importantly, these effects should be observed in metacognitive evaluations. If people have metacognitive access to the WM contents during these complex span tasks, we should find that the magnitude of confidence follows the same pattern of WM scores, meaning that both recall and confidence should be lower in the higher CL condition and affected by recency and primacy effects. We hypothesized that participants should be able, in general, to accurately monitor their performance on an item-by-item basis using subjective metacognitive evaluations. Moreover, using a measure of metacognitive sensitivity, we should show that there is a reliable relationship between performance and metacognitive evaluation. Our hypotheses and analyses were preregistered and can be found on https://osf.io/c8qfg.

## Results

Data are available on https://osf.io/hsb9e/. We tested thirty-four participants. Each participant performed 16 six-letter trials resulting in 544 trials in total (272 per CL condition). Based on typical TBRS analyses, we discarded the trials that did not achieve the criterion of 80% correct key press responses of the processing task to ensure that participants actually manage to perform the dual task. This procedure resulted in 4.6% of discarded trials, but all participants contributed data to the final analysis. In total, we analyzed 254 trials in high CL condition and 265 trials in the low CL condition. A first comparison between response times of the parity and location judgments provided a manipulation check of cognitive load. A Wilcoxon signed rank test confirmed the parity judgment effectively elicited longer RT with a median of 618 ms (IQR = 600–648) than the location judgment task with a median 445 ms (IQR = 423–488) (z = − 5.012, *p* ≤ 0.001).

Participants reported confidence for each of six positions in each trial, which makes a total of 1524 and 1590 performance-confidence observations for high and low CL conditions respectively. We computed two WM scores: item recall and strict recall as described above and participants provided metacognitive evaluations corresponding to these scoring methods: retrospective confidence of item recall and retrospective confidence of strict recall.

Data were analyzed by using R (version 4.2.0)^57^. For WM scores, we used the *lme4* package (version 1.1-30)^58^ and the function *glmer* to perform two logistic regressions through generalized linear mixed models (GLMM) that allow for analyzing binary responses. Mixed effects analyses maximize the generalizability of our results to other participants^59^. Moreover, since CL can vary across participants, mixed models allow us to control the variability that could be accounted for individual differences. We used the method proposed by Bates et al.^60^ to construct parsimonious mixed models preventing convergence problems.

For metacognitive evaluations, we also applied two ordinal logistic regressions since we used ordinal confidence scales. Thus, we used *Ordinal* package (version 2019.12-10)^61,62^. Ordinal logistic regression models aimed to evaluate whether or not magnitude of confidence for both strict recall and item recall were affected by CL and serial position. The analysis script is available on https://osf.io/pbktn/. Finally, in keeping with other studies in the literature, we performed gamma correlations to calculate metacognitive sensitivity. These correlations compare the participants’ confidence and the number of items they recall or fail to recall at each level of confidence^30,63^ (for a critique see^29^).

**Figure 1:**
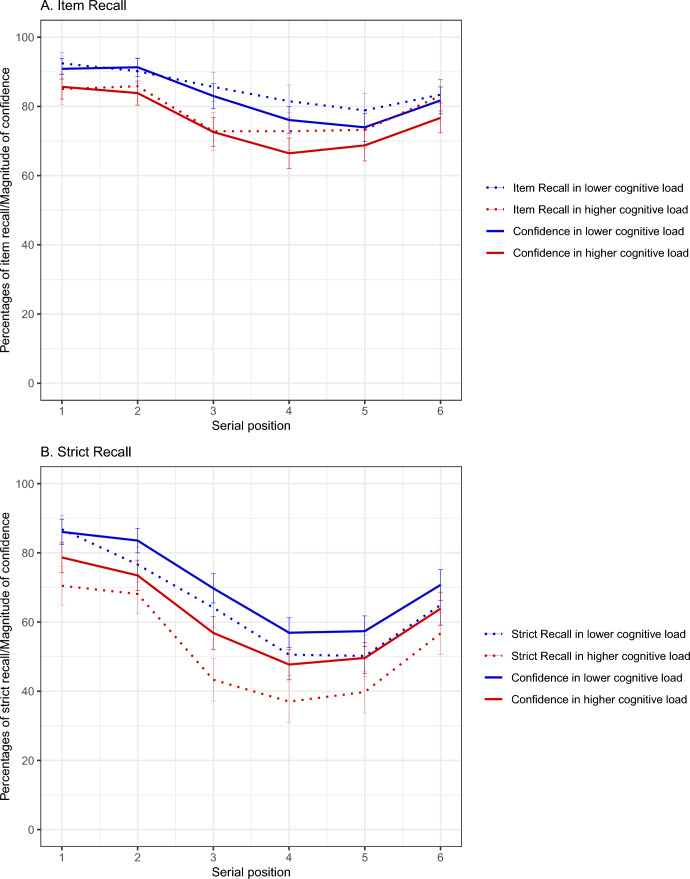
Effects of cognitive load and serial position on working memory and metacognitive evaluations. (**A**) Item recall performance and its levels of confidence as a function of serial position and cognitive load. (**B**) Strict recall performance and its levels of confidence as a function of serial position and cognitive load. Dotted lines represent the recall and solid lines the confidence. Blue indicates the lower cognitive load condition and red the higher cognitive load condition. Error bars represent 95% confidence intervals.

Figure 1 shows the mean levels of performance and retrospective confidence for each serial position and each condition of CL. The left-hand panel (a) shows the data for item scoring, and the righthand panel shows the data for strict recall scoring (b).

### Effects of cognitive load and serial position on WM scores

In line with our predictions, the binomial logistic model including CL and serial position as factors revealed significant effects on strict recall. Participants recalled fewer items in the parity judgment condition (*M* = 78.98%, *SD* = 19.06) than in the location judgment condition (*M* = 85.29%, *SD* = 15.85) *β* = − 0.57, z = − 4.88, *p* < 0.001. The same pattern occurred with the strict recall score. The participants recalled fewer items in the correct position in the parity judgment condition (*M* = 52.65%, *SD* = 26.23) than in the location judgment condition (*M* = 65.57%, *SD* = 25.27) *β* = − 0.67, z = − 5.36, *p* < 0.001.

In our examination of the effects of serial position, we utilized a comparison of models that included serial position versus models that did not include it (see Judd et al., 2017). The model incorporating serial position as a fixed effect is more robust for both item recall (Chi-square = 32.58, DF = 5, *p* < 0.001) and strict recall (Chi-square = 62.53, DF = 5, *p* < 0.001) as compared to models that do not include it. These findings are illustrated in Fig. 1A,B respectively. Our analyzes revealed that, when comparing estimated marginal means, items in first position were better recalled than those in third, fourth, fifth, and sixth position for both item recall and strict recall (all *p*-values < 0.01). Additionally, items in sixth position were better recalled than those in fourth and fifth position (all *p*-values < 0.01) on both item recall and strict recall. Results for serial position on strict and item recall were averaged over the levels of CL and Tukey method for *p*-value adjustment was applied to compare a family of six estimates. These outcomes indicate the presence of typical primacy and recency effects in WM, as items located in first and last positions were better recalled than those in the middle of the series, regardless of the type of scoring. The comparison model method did not detect global interactions between our factors for either item or strict recall.

### Effects of cognitive load and serial position on retrospective confidence

Two ordinal logistic models were employed to investigate the effects of CL and serial position on retrospective confidence of item recall and strict recall. The results indicate a significant impact of CL and serial position on both metacognitive judgments. As shown in Fig. 1A, participants exhibited lower confidence levels in item recall within the parity judgment condition (*M* = 75.34%, *SD* = 20.71) than in the location judgment condition (*M* = 82.80%, *SD* = 17.49) *β* = − 0.58, z = − 6.15, *p* < 0.001. Likewise, Fig. 1B illustrates that participants demonstrated lower confidence levels in strict recall within the parity judgment condition (*M* = 61.36%, *SD* = 26.18) compared to the location judgment condition (*M* = 70.68%, *SD* = 23.02) *β* = − 0.63, z = − 7.32, *p* < 0.001. The pattern of retrospective confidence of both WM measures followed the same pattern as the recall performance.

Furthermore, our study found that serial position has a significant effect on retrospective confidence of both item recall and strict recall. The comparison model approach revealed that models including serial position as a fixed effect were more robust than models without it for retrospective confidence of both item recall (likelihood ratio test = 218.88, *p* < 0.001) and strict recall (likelihood ratio test = 62.57, *p* < 0.001). Comparisons of estimated marginal means showed that participants exhibited more confidence for items in the first position than for items in other positions for both types of recall (all *p* < 0.03). Additionally, participants were more confident for items in the sixth position than for items in the fourth and fifth positions (all *p* < 0.01). No interaction effect was observed in any of our retrospective confidence measures.

### Metacognitive bias

On average, participants were under-confident for item recall in both the parity judgment condition (− 3.64% of difference between the confidence and the actual performance) and the location judgment condition (− 2.49% of difference between the confidence and the actual performance). Whereas for strict recall, participants were overconfident in both the parity judgment condition (8.71% of difference between the confidence and the actual performance) and the location judgment condition (5.11% of difference between the confidence and the actual performance).

### Metacognitive sensitivity

**Figure 2:**
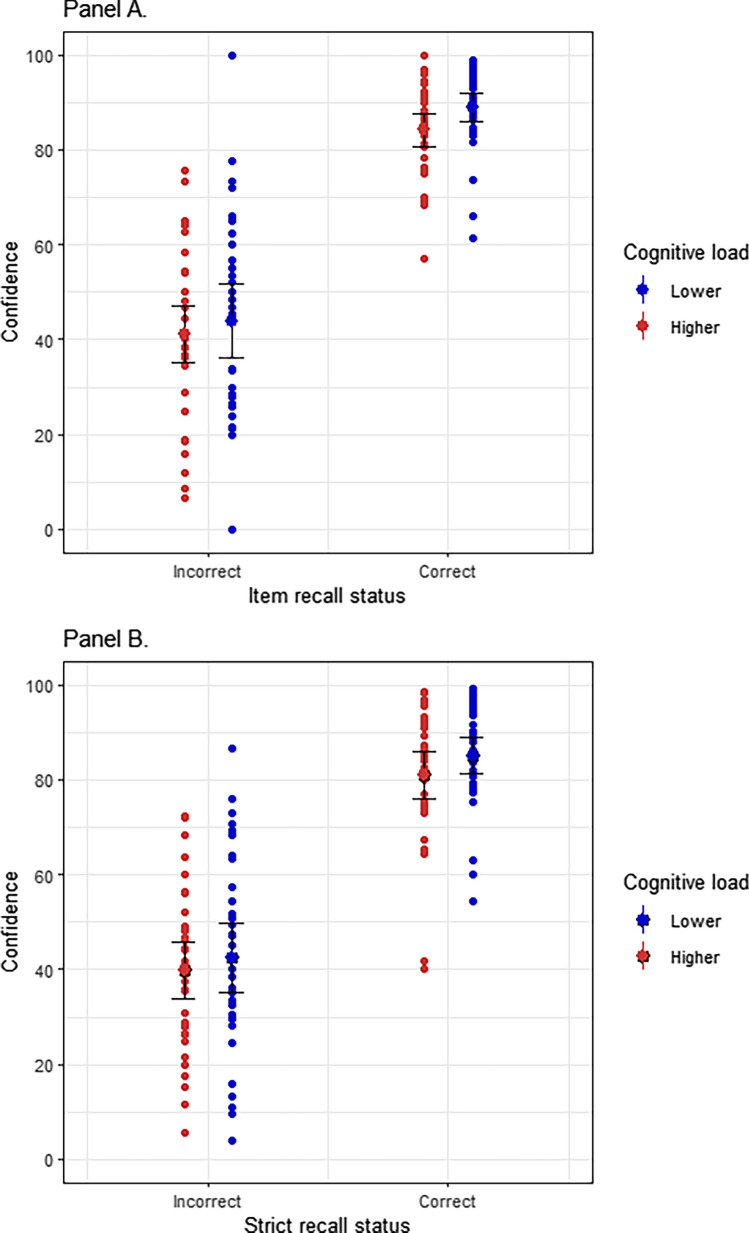
Metacognitive sensitivity. (**A**) Confidence across item recall status and cognitive load conditions. (**B**) Confidence across strict recall status and cognitive load conditions. This figure shows that confidence is influenced by recall status and cognitive load in both WM measures (item and strict). Error bars represent 95% confidence intervals.

For each participant, a Gamma correlation coefficient was calculated between recall and retrospective confidence^30^ collapsed across all serial positions. We calculated a 2 × 2 gamma, using a median split for each participant to categorize high and low confidence judgments and comparing this with the recall status (correct or incorrect) for each response. The median gamma value for item recall was 0.91 (IQR = 0.83–0.95) and for the strict recall was 0.85 (IQR = 0.78–0.91), suggesting that participants were able to accurately judge their WM performance. In support of these results and given criticism of the gamma correlations, in an analysis that we did not preregister, we performed ordinal regressions to determine if we could predict confidence with recall and CL as factors (see Fig. 2). Here we treat the recall as an independent variable, with the logic that if people make confidence judgments that are reflective of the ability to monitor performance, this should show that recall status has an impact on confidence. Our model confirms the significant effects of recall status and cognitive load on confidence (see Table 1).

**Table 1:** Recall and cognitive load as predictors of confidence.

I. Estimated coefficients for recall status as factor						
	Confidence for item recall			Confidence for strict recall		
	*β*	z	*p*	*β*	z	*p*
Correct	2.73	18.05	< 0.001	2.64	19.09	< 0.001
Incorrect	Reference level					

II. Estimated coefficients for cognitive load as factor						
	Confidence for item recall			Confidence for strict recall		
	*β*	z	*p*	*β*	z	*p*
Higher CL	− 0.42	− 4.3	< 0.001	− 0.3	− 3.88	< 0.001
Lower CL	Reference level					

All *p* values were obtained through Wald test by using the *ordinal* package in R.

## Discussion

The main goal of this study was to determine whether participants have metacognitive access to WM during a complex span task. In contrast to previous studies that primarily utilized global judgments or a probe recognition task to examine the metacognition of WM, the current study employed a local judgment approach, whereby we asked for a confidence judgment for each item retrieved, enabling the comparison of primacy and recency effects observed in serial recall. Moreover, we manipulated CL, as this produces a robust and well-documented effect on verbal WM performance. We thus compared the patterns for recall and confidence according to benchmark effects typically observed in verbal WM and targeted the awareness of the contents of WM rather than its capacity. We replicated CL and serial position effects on both WM scores. Participants recalled fewer items in the parity judgment condition (i.e., higher CL) than in the location judgment condition (i.e., lower CL). The parity judgment task requires more attentional resources to be performed; consequently, attentional resources are less available to maintain information in WM^54^. Moreover, items in first position were better recalled than those in other positions, and those in sixth position were better recalled than those in fourth and fifth positions, yielding a clear pattern of primacy and recency^36^.

Importantly, both confidence measures followed a similar pattern as recall. Participants exhibited lower levels of confidence in the higher CL condition compared to the lower CL condition. Furthermore, in alignment with previous studies^47,50^, recency and primacy effects were mirrored in metacognitive evaluations. Participants were more confident for items recalled in the first positions than for items in other positions. Likewise, they were more confident for items recalled in the sixth position than for items in the fourth and fifth positions.

These analogous patterns between retrospective confidence and WM scores suggest an association between metacognitive evaluations and recall. This association was outlined by high levels of metacognitive sensitivity as measured by Gamma, meaning that participants accurately distinguished between correctly and incorrectly recalled items in their confidence judgments. The logistic regression models also showed that confidence is a reliable predictor for strict recall and item recall, endorsing a strong association between first- and second-order performance. Altogether, our data show that participants were able to adjust their confidence according to the likelihood of their recall being correct. There are a number of theoretical proposals that explain how metacognitive judgments access memory, mostly deriving from the LTM literature, which we summarize here with reference to our task.

One first issue to consider is how our methodology impacts on metacognitive evaluations, and the extent to which our retrospective confidence judgments tap into representations held in WM. In the current experiment, the representations of the letter series may have already disappeared from WM by the time the judgments were made^23^, or at least, they were not necessary to provide a confidence response, since we provided all participants with a reminder of their recall, albeit immediately after their recall attempt. Therefore, one could consider that the metacognitive evaluations were not based on direct online awareness of the WM system, but on the retrieval experience during recall. However, some record of how recall was achieved must be accessible which influences the metacognitive evaluation.

Whether or not representations are still in WM at the moment of making the confidence judgment, our results are consistent with the first–second-order approach of metacognition^49^. Recall is the first-order behavior and retrospective judgments of confidence on WM outputs are the second-order behavior. Retrospective judgments refer to the level of confidence a participant has in being correct on a given first-order response.

As stated by Koriat^64^, local retrospective judgments, such as those employed in our study, are primarily rooted in the experience of the first-order process and are influenced by factors such as fluency and ease of processing or retrieval^41–43^. Across different cognitive domains, experience-based metacognition can be facilitated by an architecture linking first- and second-order behaviors in a reliable manner^10^. This framework theorizes the capacity for constructing meta-level representations of task difficulty to infer confidence^48,65^, with the expectation that while any factors affecting first-order performance should be reflected in confidence judgments, metacognitive sensitivity remains preserved^10^.

Our participants were tasked with retrieving information in two different conditions, one more demanding than the other, along with the recallability of certain items influenced by their position. Working memory scores clearly reflected these differences. Retrieval in the low CL condition was probably perceived as easier than in the high CL condition, resulting in higher confidence levels. In the same vein, previous research has shown that CL also affects recall time in WM tasks^66^ with shorter retrieval latencies in low compared to high CL, consistent with the view of latency as a measure of trace strength (i.e., the stronger the trace, the faster the response)^64,67,68^. Across domains, retrospective confidence judgments (i.e., second-order responses) are shown to correlate with first-order response times^10,65,69,70^. Future research should aim to measure response latency in each CL condition and serial position to gain a deeper understanding of the cues that underlie metacognition for WM, since it is possible that retrospective confidence in this task, as is shown in the literature, is related to the time taken to make the first order decision.

Metacognitive evaluations can also be based on informational cues^42^. Koriat^64^ explains that part of the overconfidence bias in retrospective judgments is due to priori beliefs about one’s own skills, the task, or the function to be tested. Interestingly, most of the studies on metacognition for WM have shown that participants tend to be overconfident (e.g.,^37,50^), and as such perhaps have pre-existing beliefs about how easy a task will be. In our study, overconfidence was found only for strict recall taking into account the item as well as its position, but not in the more liberal item recall. This suggests that participants can modulate their metacognitive evaluations in keeping with the task demands, and therefore, perhaps there is not a uniform overconfidence in WM as the literature suggests. However, this difference should be interpreted with caution as no previous studies have examined this specific question.

A question for future research is how participants are aware of the effects of CL and serial position. It remains unclear whether these two factors impact recall, which in turn affects confidence, or if they have a more direct influence on confidence, and our post-recall measure is unable to differentiate these two accounts. To differentiate between these possibilities, one approach may be to gather predictions on CL conditions and serial positions prior to retrieval, or even before encoding. This would allow us to determine whether individuals use their prior knowledge about WM and expectations to form their predictions, and thus, whether the sensitivity to CL and serial position is driven by informational cues or a direct outcome of recall performance for each CL condition and each serial position. One possibility is that serial position acts as a cue to retrieval to which participants have a direct access as suggested by previous studies. For instance, one may have information about the serial position not captured in our strict and item confidence evaluations. Imagine someone successfully retrieves the items in positions 1, 3, 4, 5, and 6, but knows that they have forgotten the item in position 2. In this case, the participants could be sure that there was missing information and be sure about the appropriate cue (the serial position), and we propose that this is akin to the feeling-of-knowing in LTM^71,72^. It would reflect a form of metacognition that was related to understanding the structure of the series and a certain ‘place’ in WM, whereby the serial position acted as a cue both for retrieval and metacognitive evaluations.

Another important area of study would involve examining how metacognitive judgments and their accuracy affect performance, as well as their potential relationship to strategy selection. Research has shown that higher levels of metacognition can influence the selection of strategies^73^. Interestingly, metacognitive beliefs and metacognitive experiences appear to have distinct impacts on strategy selection^74,75^. At present, our focus is primarily on monitoring and we set out to investigate metacognitive access to representations held in WM. Our results show that participants have the ability to discriminate between individual items recalled from WM, and confidence judgments reproduce standard CL and serial position effects. Metacognitive evaluations of WM are thus non-random. The extent to which this reflects direct metacognitive access to specific WM cues is a critical question for future research in this domain.

Given the emerging consensus that at least some aspects of post-decisional metacognition are domain-general^10,48,76^, we would not perhaps expect anything other than accurate metacognition in WM for retrospective confidence. To explore the effect of CL that impacts primarily the encoding phase, it would be necessary to further explore the tradeoff of attentional resources between maintenance, processing, and monitoring of the ongoing WM operation. As mentioned above, our experiment provides insights into metacognitive access to WM outputs and we have cautiously indicated here that retrieval fluency (as an experiential cue) may explain the patterns here. Examining informational cues would be another important question to address: for instance, do participants have an explicit model of typical serial position effects which informs their confidence judgments?

In conclusion, previous studies have demonstrated that participants are able to update and adjust their metacognitive judgments according to the type of experience during encoding WM tasks and that metacognitive judgments can map the effects of word length, phonological similarity, and serial position^37,39,40,50^. Our results contribute to the growing body of literature^37,39,40,50^ suggesting that metacognitive access to WM can occur in a similar manner to that of LTM. Additionally, the use of item-by-item judgments in our study suggests an access to WM content, going beyond investigations of WM capacity.

## Method

### Participants

Thirty-four undergraduate students between 18 and 30 years old (mean age = 20.86) voluntarily took part in this experiment. The non-inclusion criteria were: taking neuroleptics or reporting neuropsychological or psychiatric disorders. We excluded trials where participants obtained less than 80% in the processing task. The participants received course credit for participation. All methods were carried out in accordance with relevant guidelines and regulations. This study was conducted in compliance with the Code of Ethics of the World Medical Association (Declaration of Helsinki). All participants were informed about the characteristics of the study through an informed consent document that they signed before participating. The protocol was submitted to the multidisciplinary ethics committee from the Grenoble Alpes University (CERGA), from which we obtained a favorable opinion (CERGA-Avis-2021-9).

### Materials

All the tasks were coded on Open Sesame^77^ and are available in https://osf.io/s879n/. The WM task is a variation of the complex span task used in Barrouillet et al.^56^ where participants had to memorize series of 6 letters while conducting a processing task. For the memoranda, all consonants in the alphabet were used except for “W” (which is trisyllabic in French). For the processing task, stimuli were digits from 1 to 10 presented on screen in their Arabic form. Participants performed either a location judgment corresponding to the low CL condition or a parity judgment corresponding to the high CL condition. The metacognitive task encompassed two item-by-item judgments of confidence about item recall and strict recall respectively. Ordinal scales of six levels (i.e., 0, 20, 40, 60, 80, and 100) were provided for these judgments of confidence (see Fig. 2).

### Procedure

The experimental phase consisted of 16 series, each composed of 6 to-be-remembered letters interleaved with a processing task. Each series started with a 750 ms fixation signal (an asterisk) centered on the screen that was replaced by the first letter to be remembered. Each letter was displayed on the screen for 1500 ms followed by a delay of 500 ms. After each letter, the 6 digits to be processed (i.e., distractors) appeared sequentially on screen, with each digit appearing either in the lower or in the upper part of the screen. They were displayed for 711 ms followed by a delay of 356 ms. Hence, the time available to process each digit was 1067 ms, resulting in a constant inter-letter interval of 6402 ms. For the high CL condition, participants had to judge the parity of the digits. They had to verbally report “odd” (in French, “impair”) and to press “S” simultaneously if the digit was odd, or say “even” (“pair”) and press “L” simultaneously if the digit was even. For the low CL condition, the participants judged the stimuli’s location by either pressing “S” and saying aloud “down” (in French, “bas”) if the stimuli were located on the lower part of the screen or pressing “L” and saying aloud “up” (“haut”) if they were located on the upper part of the screen. At the end of each series, the word Recall appeared at the center of screen to inform the participants that they had to recall the letters in the correct order by using the keyboard. Participants were told to guess if they did not remember a letter.

After the recall phase of each series, participants were re-presented their 6 recalled letters all at once, in the order given, and were asked to rate, one by one, their confidence in (a) the item recall by indicating the degree of confidence that the letter actually appeared in the series (in French “à quel point êtes-vous certain que la lettre ‘X’ est apparue dans cette série ?”), and (b) the strict recall by indicating the degree of confidence that the letter actually appeared in the series and that it had been recalled in the correct position (in French “à quel point êtes-vous certain que la lettre ‘X’ est apparue dans cette série ET que vous l'avez rappelée à la bonne position ?”).

**Figure 3:**
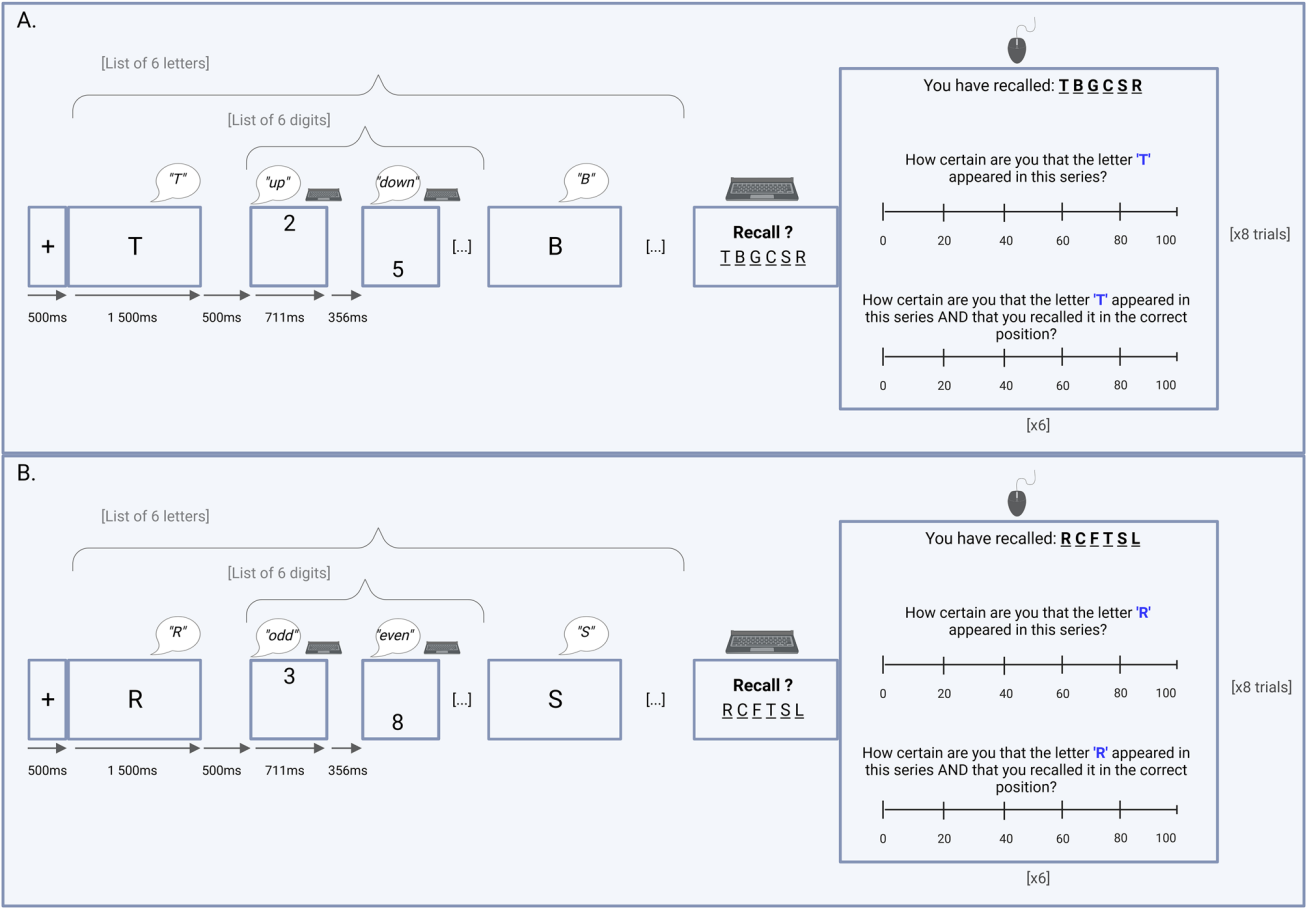
Illustration of the structure of the task. (**A**) Complex span task under local judgment condition. This represents the low cognitive load scenario. Participants were required to respond with a “L” key press when the number appeared on the upper side of the screen, simultaneously verbalizing “*up”*; similarly, a “S” key press was prompted when the number emerged on the lower side, accompanied by the verbalization “*down”*. (**B**) Complex span task in parity judgment condition. This represents the higher cognitive load scenario. Participants were instructed to press “S” for odd digits while verbalizing “*odd”*, and to press “L” for even digits while verbalizing “*even”*. In both conditions, participants had to recall 6 letters per series and to rate their confidence in the letters’ appearance and the positional recall.

The series were presented in blocks of CL, each block containing four series. This resulted in two blocks of high CL and two blocks of low CL administered in a counterbalanced fashion. Each participant produced 96 retrospective judgments of confidence (8 series × 2 conditions × 6 letters) (see Fig. 3). Lastly, at the end of each block, participants were asked to rate their level of confidence in processing task performance in order to keep them motivated. For instance, at the end of the block of parity judgment, the following question appeared “How successfully do you consider your performance on the PARITY judgment task? [0 = 0% success, 100 = 100% success]. Enter a number between 0 and 100” (in French “à quel point considérez-vous avoir réussi la tâche de jugement de PARITE ? [0 = 0% de réussite, 100 = 100% de réussite]. Saisissez un chiffre entre 0 et 100”).

The experimental phase was preceded by a training phase. First, participants trained themselves in the processing task only (thirty stimuli for the parity and the location judgment tasks respectively). Then, they trained themselves in maintenance tasks with a series of simple spans of 6 letters. Then, the two item-by-item judgments of confidence were performed after the simple spans. Finally, participants trained themselves with the entire task meaning that they performed two trials identical to the experimental phase: memory task, processing task, recall, and judgments of confidence. This training was performed with exactly the same temporal conditions as in the experimental phase.

## Data Availability

All raw data have been deposited online and can be freely accessed on https://osf.io/n3k2z/. Pre-registration can be accessed on https://osf.io/c8qfg.

## References

[1] Beer JS, John OP, Scabini D, Knight RT (2006). Orbitofrontal cortex and social behavior: Integrating self-monitoring and emotion-cognition interactions. J. Cogn. Neurosci..

[2] Kessel R (2014). Metacognitive monitoring of attention performance and its influencing factors. Psychol. Res..

[3] Kramarski B, Mevarech ZR (2003). Enhancing mathematical reasoning in the classroom: The effects of cooperative learning and metacognitive training. Am. Educ. Res. J..

[4] Nozari N, Novick J (2017). Monitoring and control in language production. Curr. Dir. Psychol. Sci..

[5] Simon DA, Bjork RA (2001). Metacognition in motor learning. J. Exp. Psychol. Learn. Mem. Cogn..

[6] Song C (2011). Relating inter-individual differences in metacognitive performance on different perceptual tasks. Conscious. Cogn..

[7] Yeung N, Summerfield C (2012). Metacognition in human decision-making: Confidence and error monitoring. Philos. Trans. R. Soc. B.

[8] Vaccaro AG, Fleming SM (2018). Thinking about thinking: A coordinate-based meta-analysis of neuroimaging studies of metacognitive judgements. Brain Neurosci. Adv..

[9] Dunlosky J, Bjork RA (2008). Handbook of Metamemory and Memory.

[10] Mazancieux, A. *et al.* A common conceptual space for metacognition across and within domains. *Manuscript submitted for publication* (2022).

[11] Logie RH, Camos V, Cowan N (2021). Working Memory: State of the Science.

[12] Baddeley AD, Hitch G, Allen R, Logie RH, Camos V, Cowan N (2021). A multicomponent model of working memory. Working Memory.

[13] Barrouillet P, Camos V, Logie RH, Camos V, Cowan N (2021). The time-based resource-sharing model of working memory. Working Memory.

[14] Cowan N, Morey CC, Naveh-Benjamin M, Logie R, Camos V, Cowan N (2021). An embedded-processes approach to working memory: How is it distinct from other approaches, and to what ends?. Working Memory.

[15] Nelson, T. O. & Narens, L. Metamemory: A theoretical framework and new findings. In *Psychology of Learning and Motivation* (ed G.H. Bower) **26**, 125–173 (New York: Academic Press, 1990).

[16] Nelson TO, Dunlosky J (1991). When people’s judgments of learning (JOLs) are extremely accurate at predicting subsequent recall: The “delayed-JOL effect”. Psychol. Sci..

[17] Dunlosky J, Nelson TO (1992). Importance of the kind of cue for judgments of learning (JOL) and the delayed-JOL effect. Mem. Cogn..

[18] Dunlosky J, Nelson TO (1994). Does the sensitivity of judgments of learning (JOLs) to the effects of various study activities depend on when the JOLs occur?. J. Mem. Lang..

[19] Baddeley AD, Andrade J (2000). Working memory and the vividness of imagery. J. Exp. Psychol. Gen..

[20] Maniscalco B, Lau H (2015). Manipulation of working memory contents selectively impairs metacognitive sensitivity in a concurrent visual discrimination task. Neurosci. Conscious..

[21] Van den Berg R, Yoo AH, Ma WJ (2017). Fechner’s law in metacognition: A quantitative model of visual working memory confidence. Psychol. Rev..

[22] Shea N, Frith CD (2019). The global workspace needs metacognition. Trends Cogn. Sci..

[23] Cowan N (2010). The magical mystery four: How is working memory capacity limited, and why?. Curr. Dir. Psychol. Sci..

[24] Cowan, N. What are the differences between long-term, short-term, and working memory? In *Progress in Brain Research: The Essence of Memory* (eds. Sossin, W., Lacaille, J.-C., Castellucci, V. F. & Belleville, S.) vol. 169 323–338 (Elsevier B.V, 2008). 10.1016/S0079-6123(07)00020-9 PMC2657600 18394484

[25] Oberauer K, Farrell S, Jarrold C, Lewandowsky S (2016). What limits working memory capacity?. Psychol. Bull..

[26] Bartsch LM, Oberauer K (2021). The effects of elaboration on working memory and long-term memory across age. J. Mem. Lang..

[27] Oberauer K, Logie RH, Camos V, Cowan N (2021). Towards a theory of working memory: From metaphors to mechanisms. Working Memory.

[28] Schraw G (2009). A conceptual analysis of five measures of metacognitive monitoring. Metacogn. Learn..

[29] Fleming SM, Lau HC (2014). How to measure metacognition. Front. Hum. Neurosci..

[30] Nelson TO (1984). A comparison of current measures of the accuracy of feeling-of-knowing predictions. Psychol. Bull..

[31] Murphy MD, Sanders RE, Gabriesheski AS, Schmitt FA (1981). Metamemory in the aged. J. Gerontol..

[32] Flavell JH, Friedrichs AG, Hoyt JD (1970). Developmental changes in memorization processes. Cogn. Psychol..

[33] Ebbinghaus H (1913). Memory: A Contribution to Experimental Psychology.

[34] Deese J, Kaufman RA (1957). Serial effects in recall of unorganized and sequentially organized verbal material. J. Exp. Psychol..

[35] Atkinson RC, Shiffrin RM, Spence KW, Spence JT (1968). Human memory: A proposed system and its control processes. The Psychology of Learning and Motivation: II.

[36] Glanzer M (1968). Storage mechanisms in free recall. Trans. N. Y. Acad. Sci..

[37] Bunnell JK, Baken DM, Richards-Ward L (1999). The effect of age on metamemory for working memory. N. Z. J. Psychol..

[38] Baddeley AD, Hitch GJ, Bower GH (1974). Working memory. The Psychology of Learning and Motivation.

[39] Bertrand JM, Moulin CJA, Souchay C (2017). Short-term memory predictions across the lifespan: Monitoring span before and after conducting a task. Memory.

[40] Bertrand JM (2019). In the here and now: Short term memory predictions are preserved in Alzheimer’s disease. Cortex.

[41] Liberman V (2004). Local and global judgments of confidence. J. Exp. Psychol. Learn. Mem. Cogn..

[42] Koriat, A., Nussinson, R., Bless, H. & Shaked, N. Information-based and experience-based metacognitive judgments. In *Handbook of Metamemory and Memory* (eds. Dunlosky, J. & Bjork, R. A.), 117–135 (Psychology Press, 2008). 10.4324/9780203805503.ch7.

[43] Händel M, de Bruin ABH, Dresel M (2020). Individual differences in local and global metacognitive judgments. Metacogn. Learn..

[44] Schraw G, Kuch F, Gutierrez AP (2013). Measure for measure: Calibrating ten commonly used calibration scores. Learn. Instr..

[45] Unsworth N, Engle RW (2007). On the division of short-term and working memory: An examination of simple and complex span and their relation to higher order abilities. Psychol. Bull..

[46] Jacobs C, Silvanto J (2015). How is working memory content consciously experienced? The ‘conscious copy’ model of WM introspection. Neurosci. Biobehav. Rev..

[47] Reyes G, Sackur J (2018). Introspection during short-term memory scanning. Q. J. Exp. Psychol..

[48] Mazancieux A, Fleming SM, Souchay C, Moulin CJA (2020). Is there a G factor for metacognition? Correlations in retrospective metacognitive sensitivity across tasks. J. Exp. Psychol. Gen..

[49] Fleming SM, Dolan RJ, Frith CD (2012). Metacognition: Computation, biology and function. Philos. Trans. R. Soc. B.

[50] Sahar T, Sidi Y, Makovski T (2020). A metacognitive perspective of visual working memory with rich complex objects. Front. Psychol..

[51] Barrouillet P, Bernardin S, Camos V (2004). Time constraints and resource sharing in adults’ working memory spans. J. Exp. Psychol. Gen..

[52] Barrouillet P, Camos V, Osaka N, Logie RH, D’Esposito M (2007). The time-based resource-sharing model of working memory. The Cognitive Neuroscience of Working Memory.

[53] Portrat S, Barrouillet P, Camos V (2008). Time-related decay or interference-based forgetting in working memory?. J. Exp. Psychol. Learn. Mem. Cogn..

[54] Barrouillet P, Portrat S, Camos V (2011). On the law relating processing to storage in working memory. Psychol. Rev..

[55] Barrouillet P, Portrat S, Vergauwe E, Diependaele K, Camos V (2011). Further evidence for temporal decay in working memory: Reply to Lewandowsky and Oberauer (2009). J. Exp. Psychol. Learn. Mem. Cogn..

[56] Barrouillet P, Bernardin S, Portrat S, Vergauwe E, Camos V (2007). Time and cognitive load in working memory. J. Exp. Psychol. Learn. Mem. Cogn..

[57] R Core Team. A Language and Environment for Statistical Computing. (2022).

[58] Bates, D., Mächler, M., Bolker, B. & Walker, S. Fitting linear mixed-effects models using lme4. *J. Stat. Softw.* **67** 1–48 (2015).

[59] Judd CM, McClelland GH, Ryan CS (2017). Data Analysis: A Model Comparison Approach to Regression, ANOVA, and Beyond.

[60] Bates, D., Kliegl, R., Vasishth, S. & Baayen, H. Parsimonious Mixed Models. Preprint at http://arxiv.org/abs/1506.04967 (2018).

[61] Christensen, R. H. B. Cumulative Link Models for Ordinal Regression with the R Package ordinal. 46 (2018).

[62] Venables WN, Ripley BD (2002). Modern Applied Statistics with S.

[63] Nelson TO (1996). Gamma is a measure of the accuracy of predicting performance on one item relative to another item, not of the absolute performance on an individual item comments on Schraw (1995). Appl. Cogn. Psychol..

[64] Koriat A, Zelazo PD, Moscovitch M, Thompson E (2007). Metacognition and consciousness. The Cambridge Handbook of Consciousness.

[65] Koriat A (2012). The self-consistency model of subjective confidence. Psychol. Rev..

[66] Nilsson EJ, Aust ML, Engström J, Svanberg B, Lindén P (2018). Effects of cognitive load on response time in an unexpected lead vehicle braking scenario and the detection response task (DRT). Transp. Res. Part F Traffic Psychol. Behav..

[67] Murdock BB (1974). Human Memory: Theory and Data.

[68] Madigan S, Neuse J, Roeber U (2000). Retrieval latency and “at-risk” memories. Mem. Cogn..

[69] Oppenheimer DM (2008). The secret life of fluency. Trends Cogn. Sci..

[70] Rahnev D (2020). The confidence database. Nat. Hum. Behav..

[71] Schacter DL (1983). Feeling of knowing in episodic memory. J. Exp. Psychol. Learn. Mem. Cogn..

[72] Souchay C, Isingrini M, Espagnet L (2000). Aging, episodic memory feeling-of-knowing, and frontal functioning. Neuropsychology.

[73] Bebko JM, McMorris CA, Metcalfe A, Ricciuti C, Goldstein G (2014). Language proficiency and metacognition as predictors of spontaneous rehearsal in children. Can. J. Exp. Psychol./Revue Can. Psychol. Exp..

[74] Hu X, Luo L, Fleming SM (2019). A role for metamemory in cognitive offloading. Cognition.

[75] Grinschgl S, Meyerhoff HS, Schwan S, Papenmeier F (2021). From metacognitive beliefs to strategy selection: Does fake performance feedback influence cognitive offloading?. Psychol. Res..

[76] Lee ALF, Ruby E, Giles N, Lau H (2018). Cross-domain association in metacognitive efficiency depends on first-order task types. Front. Psychol..

[77] Mathôt S, Schreij D, Theeuwes J (2012). OpenSesame: An open-source, graphical experiment builder for the social sciences. Behav. Res..

